# Supplementary material for: Comparative Analysis of Transposable Elements in Strawberry Genomes of Different Ploidy Levels
Source: Int J Mol Sci. 2023 Nov 29;24(23):16935. doi: 10.3390/ijms242316935 (PMC10706760; doi:10.3390/ijms242316935)
Supplement: Supplementary file 1 [file ijms-24-16935-s001.zip › Supplementary Figure S1-S3.docx]

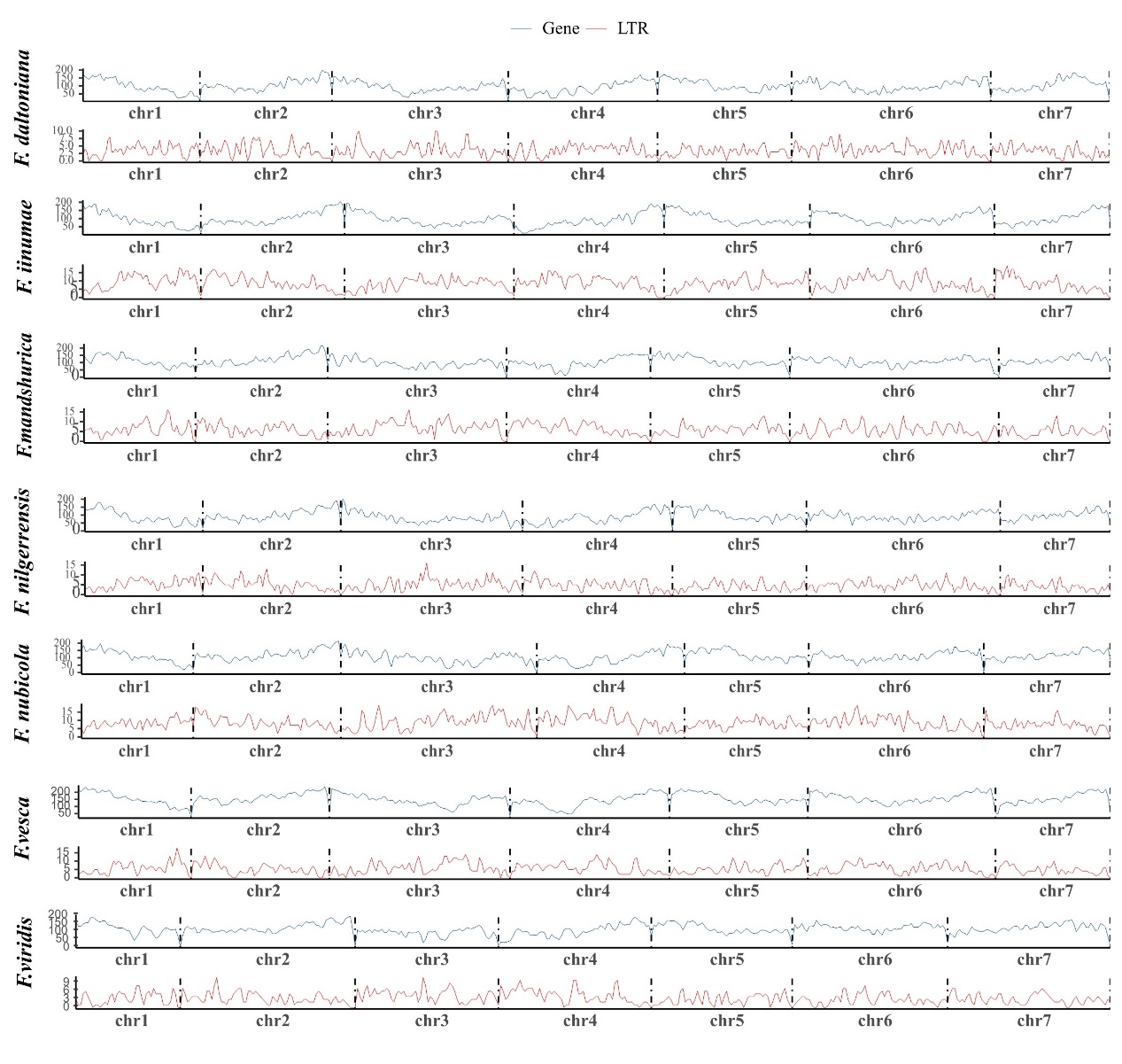


**Figure S1.** Distribution of genes and LTR-RTs on the pseudochromosomes of diploid strawberry species. Blue line is the distribution of genes, red line is the distribution of LTR-RTs.


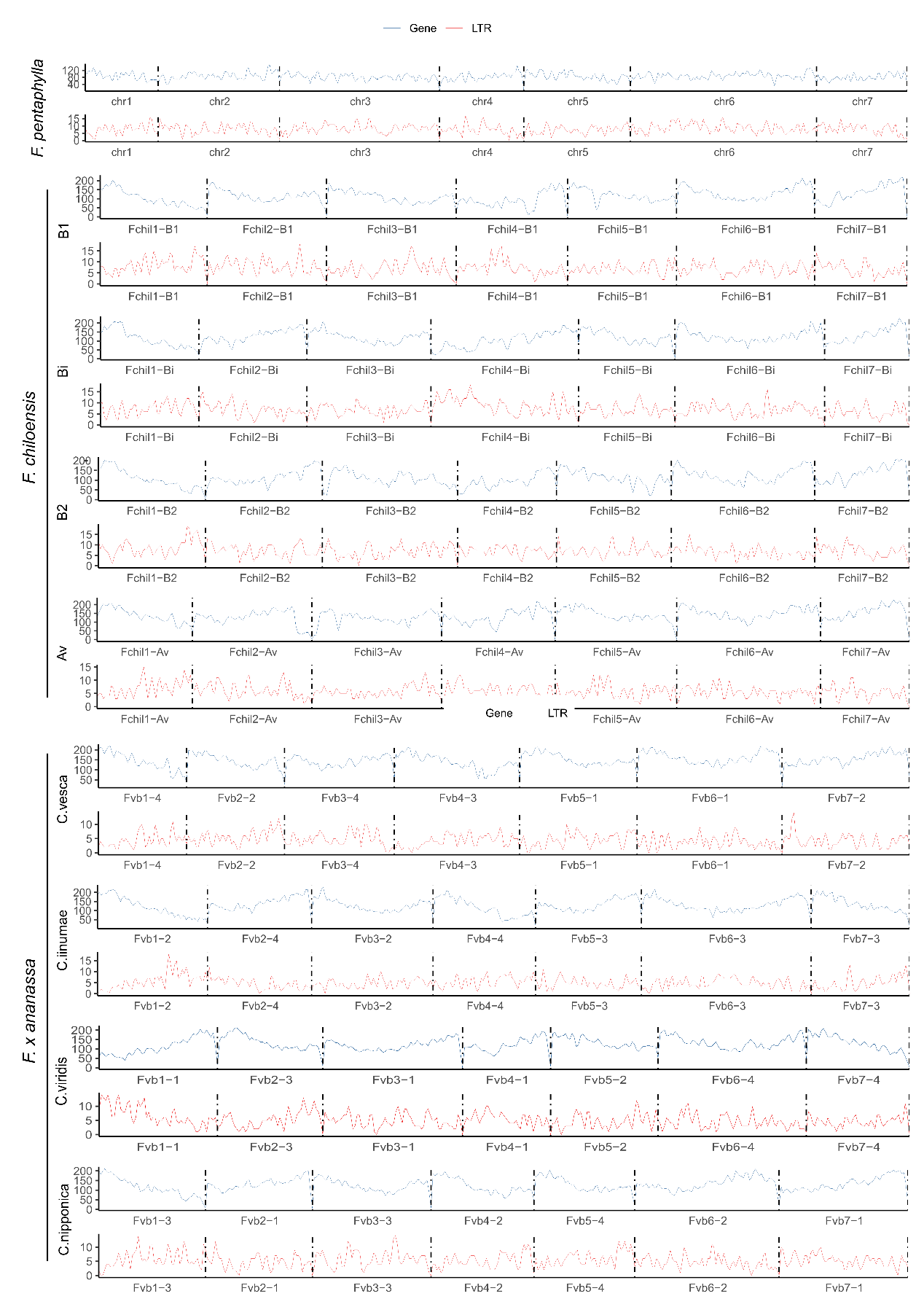


**Figure S2.** Distribution of genes and LTRs on the pseudochromosomes of polyploid strawberry species. Blue line is the distribution of genes, red line is the distribution of LTR-RTs.


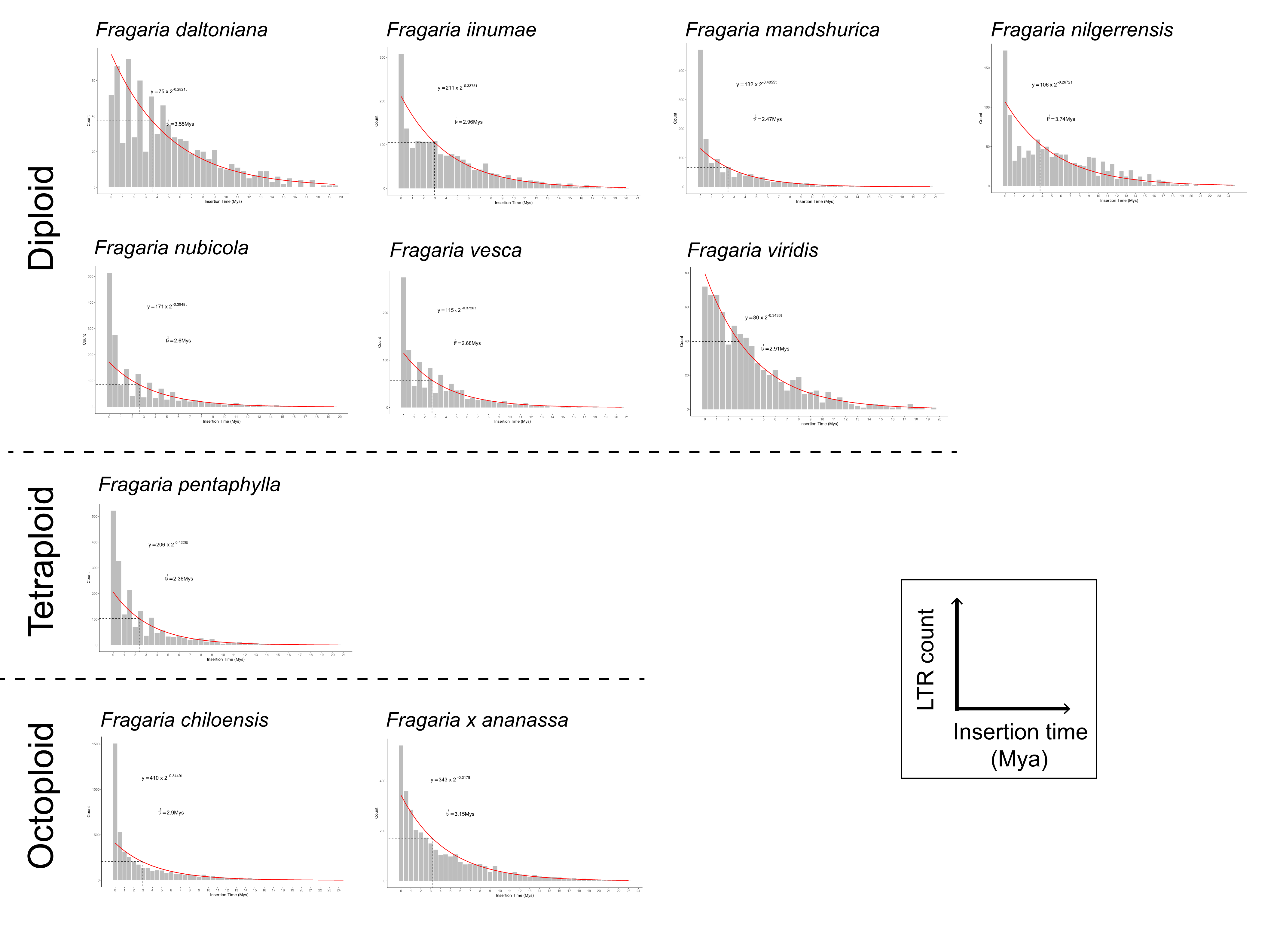


**Figure S3.** Distribution of LTR-RTs insertion times. Estimated insertion times were divided into bins of 500,000 years.
